# Supplementary material for: Metformin hydrolase is a recently evolved nickel-dependent heteromeric ureohydrolase
Source: Nat Commun. 2024 Sep 14;15:8045. doi: 10.1038/s41467-024-51752-5 (PMC11399263; doi:10.1038/s41467-024-51752-5)
Supplement: Supplementary file 1 — Supplementary Information [file 41467_2024_51752_MOESM1_ESM.pdf]

Supplementary Information for

Metformin hydrolase is a recently evolved nickel-dependent heteromeric ureohydrolase

M. Sinn[1*], L. Riede[1], J. R. Fleming[2], D. Funck[1], H. Lutz[2], A. Bachmann[2], O. Mayans[2,3] and J. S. Hartig[1,3*]

[1] Department of Chemistry, University of Konstanz, Konstanz, Germany

[2] Department of Biology, University of Konstanz, Konstanz, Germany

[3] Konstanz Research School Chemical Biology (KoRS-CB), University of Konstanz, Konstanz, Germany

Content:

- Supplementary Figure 1: Schematic representation of the expression constructs for MefH and DmgH

- Supplementary Figure 2: Denaturing polyacrylamide gel with purified *Pm*MefH

- Supplementary Figure 3: Calibration of guanylurea quantification by LC-MS

- Supplementary Figure 4: Sequence Alignment of MefH and DmgH

- Supplementary Figure 5: 6x His-PmMefH purified after expression in the presence of different metal ions and expression and purification of *Pm*MefHα and *Pm*MefHβ

- Supplementary Figure 6: Metal dependency of DmgH activity

- Supplementary Figure 7: ICP-OES analysis of the DmgH metal content

- Supplementary Figure 8: *An*MefH and DmgH kinetics in HEPES buffer

- Supplementary Figure 9: Size exclusion chromatography and dynamic light scattering of *An*MefH

- Supplementary Figure 10: Urea bound in the MefHα active site

- Supplementary Figure 11: Biuret binding site of MefHα.

- Supplementary Figure 12: Phylogenetic tree of MefH and related sequences

- Supplementary Figure 13: Analysis of the DmgH from *Hyphomicrobium*

- Supplementary Figure 14: Expression of MefH/DmgH mixed heteromers and DmgH variants

- Supplementary Figure 15: Enzymatic activity of DgmH variant $T_{289}S$.

- Supplementary Figure 16: Linearity of guanylurea and urea production over time

- Supplementary Figure 17: Uncropped picture of the denaturing polyacrylamide gel gel shown in Supplementary Figure 2.

- Supplementary Figure 18: Uncropped picture of the denaturing polyacrylamide gel gel shown in Supplementary Figure 14.

- Supplementary Table 1: Peptide mass fingerprint analysis of purified *Pm*MefH

- Supplementary Table 2: Compounds of the substrate screen

- Supplementary Table 3: X-ray diffraction data collection and refinement statistics

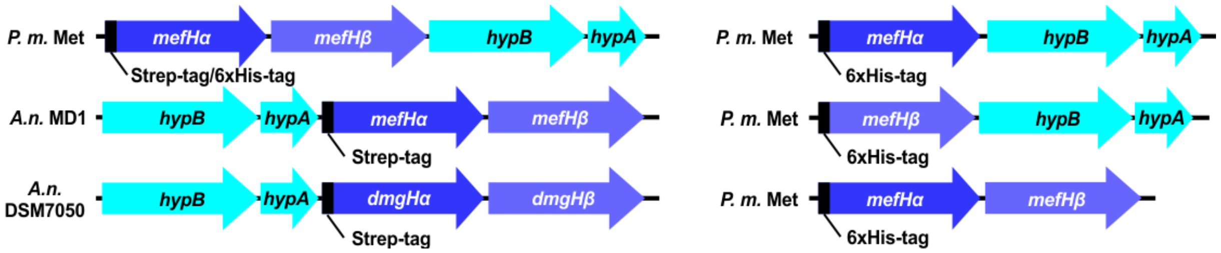

Supplementary Figure 1: Schematic representation of the expression constructs for MefH and DmgH. In each case, only one ureohydrolase family protein was 6x His- or Strep-tagged for purification. Note that the operon was shuffled for *P. mendocina* (*P.m.*) MET, whereas the constructs with genes from *Aminobacter niigataensis (A.n.)* strains DSM7050 and MD1 are in the native order.

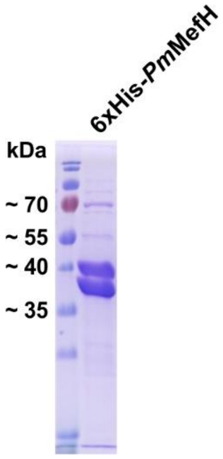

Supplementary Figure 2: Denaturing polyacrylamide gel with purified *Pm*MefH. 6x His-PmMefH was purified by nickel affinity chromatography. Two bands were observed at the expected sizes of 6xHis-MefHα (42 kDa) and MefHβ (37 kDa). An uncropped version of the gel image can be found in Supplementary Figure 17.

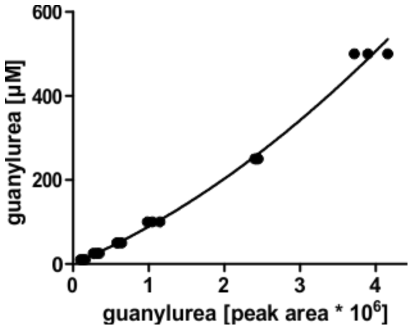

Supplementary Figure 3: Calibration of guanylurea quantification by LC-MS. Standards in reaction buffer containing guanylurea and metformin a combined concentration of 2 mM were measured in technical triplicates. Data were fitted with a polynomic regression (solid line, R^2= 0.9931). The resulting equation was used to calculate guanylurea concentrations of the enzyme assay samples. Source data are provided as a Source Data file.

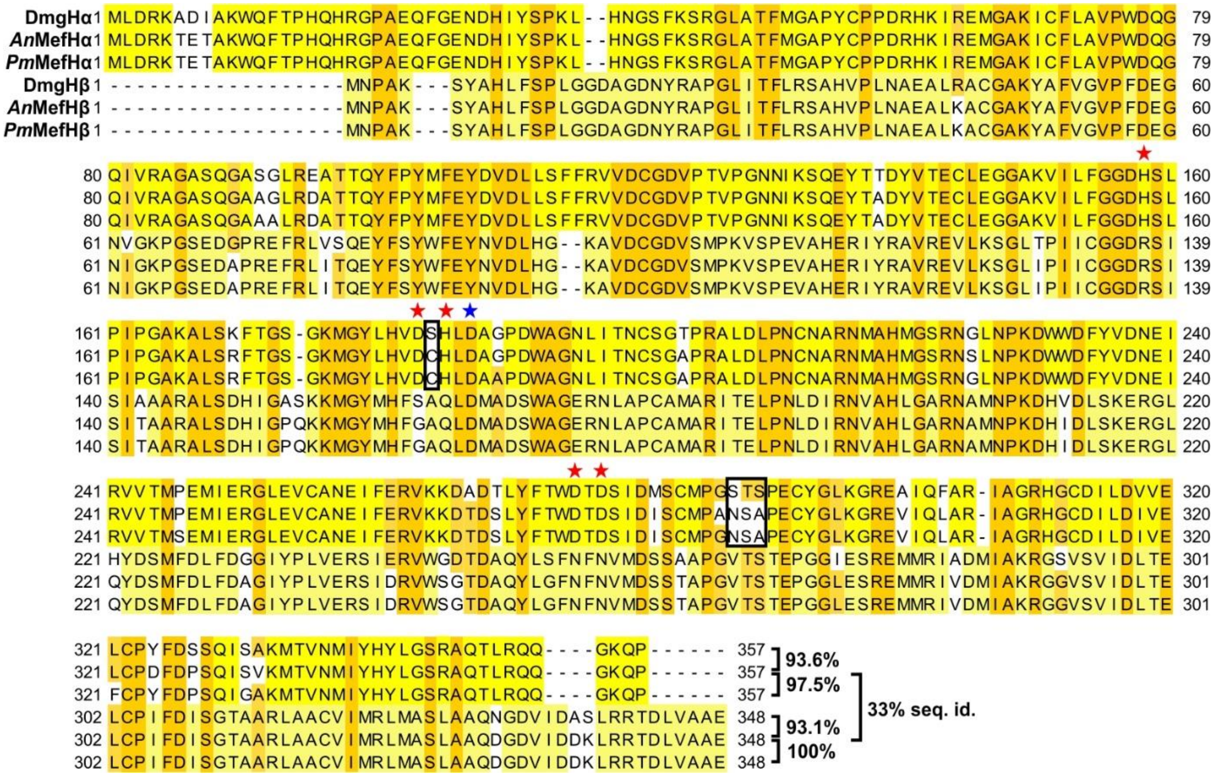

Supplementary Figure 4: Sequence Alignment of MefH and DmgH subunits. Clustal omega was used to generate a multiple sequence alignment of all six subunits of *An*MefH, *Pm*MefH and DmgH. Residues conserved in all proteins are highlighted in dark orange, over 50% conservation is highlighted in light orange. Residues that are completely conserved in the corresponding subunits are highlighted in yellow. Residues participating in metal binding (as inferred from the crystal structure and alignment with GdmH, not shown for reasons of clarity) are marked with asterisks (red: mutated in β-subunits, blue: conserved throughout all proteins). Residues changed in the mutational studies are marked with a black box.

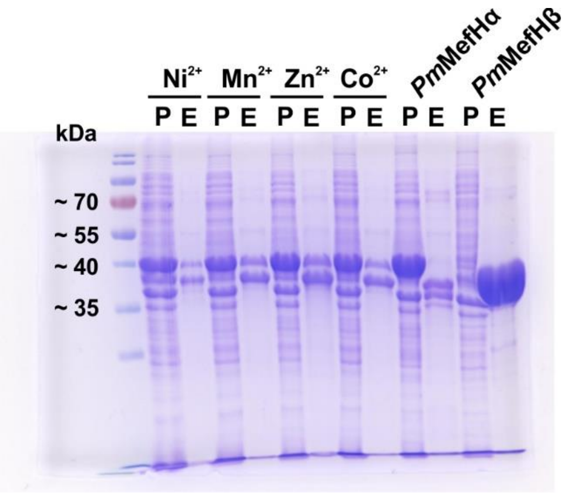

Supplementary Figure 5: 6xHis-*Pm*MefH purified after expression in the presence of different metal ions. 6xHis-*Pm*MefH was expressed in the presence of 50 µM Ni^{2+}, Mn^{2+}, Zn^{2+} or $Co^{2+}$and purified by nickel affinity chromatography. Insoluble fractions after lysis (P) and purified enzymes (E) were analyzed by denaturing polyacrylamide gel electrophoresis.

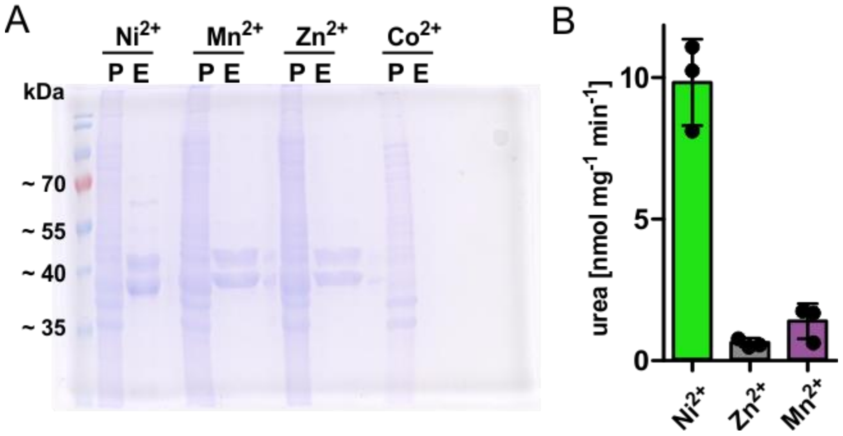

Supplementary Figure 6: Metal dependency of DmgH activity. A Denaturing polyacrylamide gel of purified Strep-DmgH after expression in the presence of different metals. Strep-DmgH was expressed in the presence of 100 µM Ni^{2+}, Mn^{2+}, Zn^{2+} or Co^{2+} and purified by nickel affinity chromatography. Insoluble fraction after lysis (P) and purified enzyme (E) were loaded. Please note that expression in the presence of Co^{2+} failed. Repetition of expression resulted in poor yields of enzyme. In light of the results with *Pm*MefH, that was expressed but inactive in the presence of Co^{2+}, we decided to stop the experiments. **B** Urea production rates were determined for purified DmgH expressed with the annotated metals. Columns represent the average of triplicates and consistent results were obtained with independent preparations (n=3; error bars, s.d.). Source data are provided as a Source Data file.

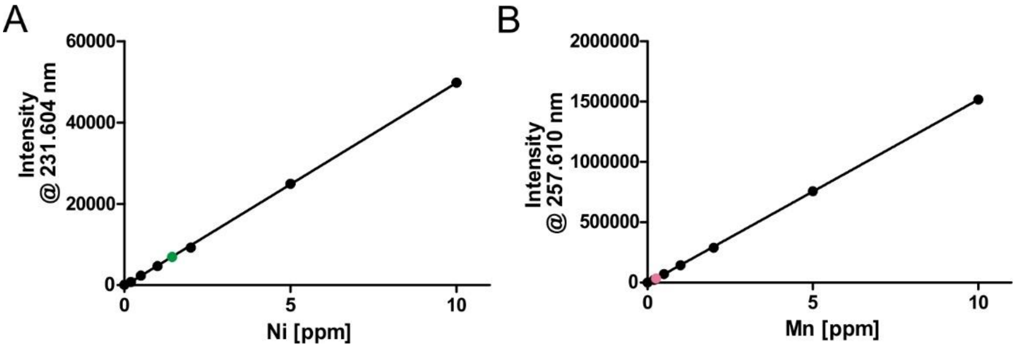

Supplementary Figure 7: ICP-OES analysis of the DmgH metal content. DmgH was expressed in medium containing 0.5 mM Ni^{2+}. The cells were lysed in virtually Ni^{2+}-free buffer and DmgH was purified by Strep-Tactin affinity chromatography followed by a buffer exchange into enzyme buffer on a PD-Midi desalting column. Metal standards containing nickel (Ni) (**A**) and manganese (Mn) (**B**) (Agilent) were measured in enzyme buffer (black dots). Data were fitted with a linear regression (solid line, R^2 = 0.9998 and 0.9999, respectively) and were used to calculate the nickel (green dot) (**A**) and manganese (magenta dot) (**B**) content of the protein samples. Data points represent the average of technical triplicates and consistent results were obtained with independent preparations. (n=3; error bars, s.d.). Please note that error bars were too small to be visualized on the scale of the graph. Source data are provided as a Source Data file.

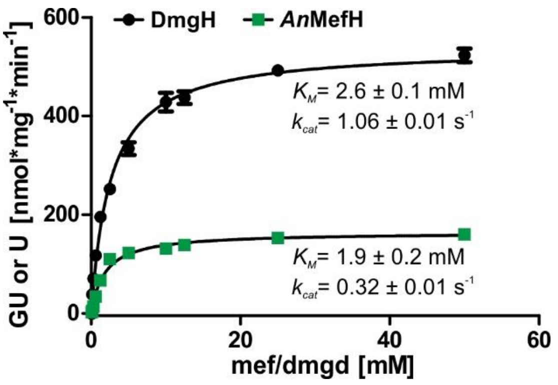

Supplementary Figure 8: AnMefH and DmgH kinetics in HEPES buffer. Urea (U) or guanylurea (GU) production at different substrate concentrations (metformin (mef) or dimethylguanidine (dmgd)) in HEPES buffer at pH 8 was measured to determine Michaelis constant K_M and maximal reaction rate (v_{max}) for DmgH (black circles) and *An*MefH (green squares). Data were fitted using Michaelis-Menten equation (R^2= 0.9930; R^2= 0.9793, respectively). Data points represent the average of triplicates and consistent results were obtained with independent preparations (n=3; error bars, s.d.). Source data are provided as a Source Data file.

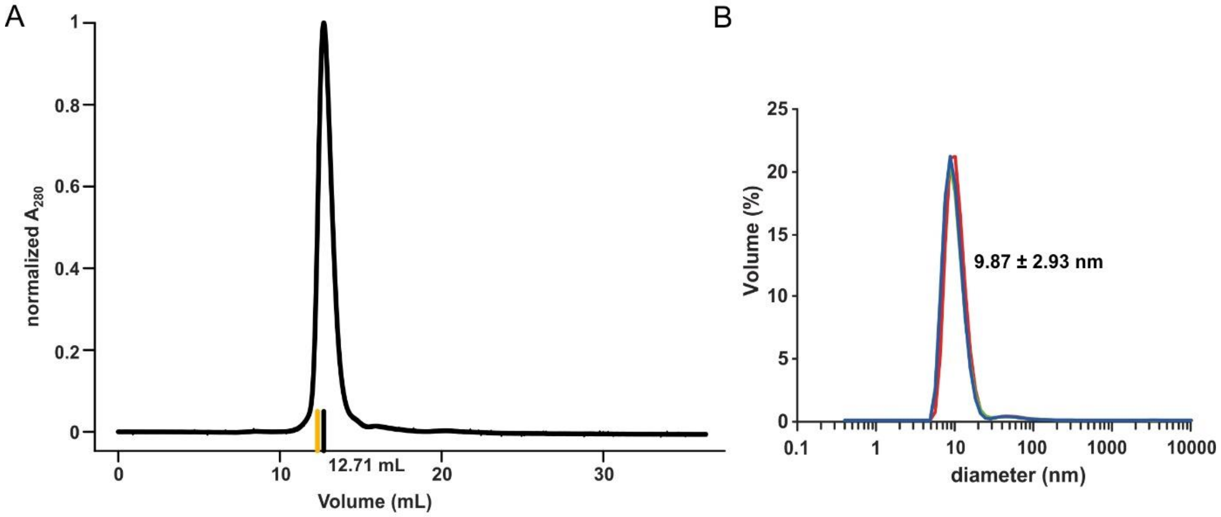

Supplementary Figure 9: Size exclusion chromatography and dynamic light scattering of *An*MefH. A Analytical size exclusion of *An*MefH. The protein size based on the elution volume of 12.71ml (black bar) was calculated to be 196 kDa. The expected volume for a 240 kDa protein corresponding to the size of the hexamer would be 12.32ml (orange bar). **B** Size distribution of purified *An*MefH determined by dynamic light scattering. Assuming spherical particles, a diameter of roughly 10 nm was calculated, which corresponds well to the diameter of the hexamer estimated from the crystal structure with UCSF Chimera[1]. Three consecutive measurements with at least two minutes pause between the runs are shown.

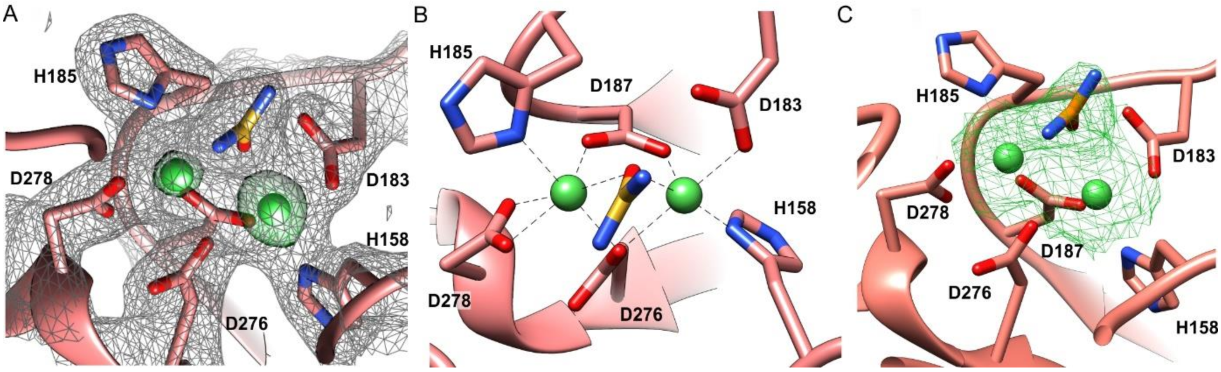

Supplementary Figure 10: Detailed views of the MefHα active site. A (2Fo-Fc)$_{\alpha calc}$ Electron density map contoured at 1σ (gray mesh) around the MefHα active site with the bound urea (gold). Around the metal ions, a (2Fo-Fc)$_{\alpha calc}$ electron density map contoured at 7σ (green mesh) is additionally displayed. **B** Coordination of the Ni^{2+} ions (green spheres) by the respective amino acids and urea (golden). The coordination was calculated in UCSF Chimera [1]. **C** Omit electron density map of the nickel ions and the urea bound in the active site contoured at 1σ level (green mesh). Model coordinates and experimental diffraction data have been deposited with the Protein Data Bank under accession code 8RYI (https://doi.org/10.2210/pdb8ryi/pdb).

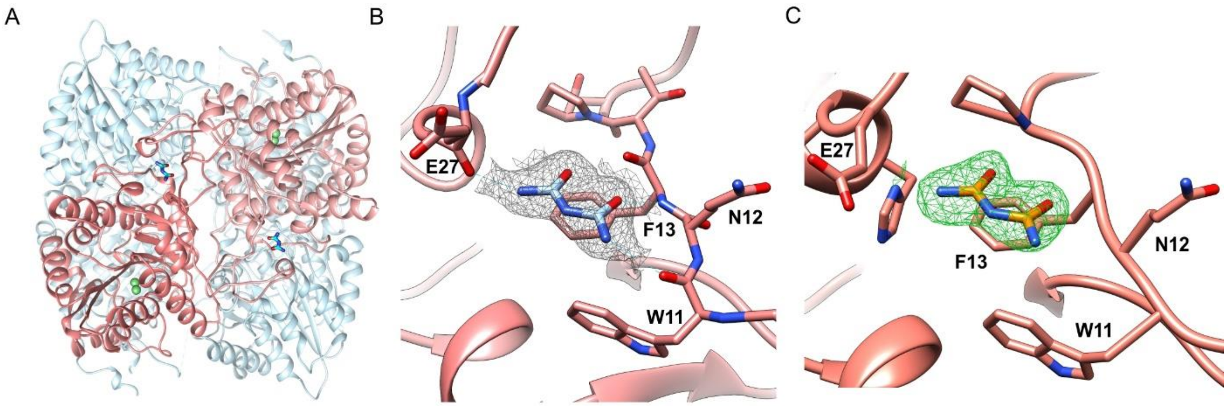

Supplementary Figure 11: Biuret binding site of MefH. A Global view of the biuret binding site in MefH. Biuret (cyan) is bound at the N-terminus of MefHα (red ribbons). The biuret binding site is distant from the active center with the Ni^{2+} ions (green spheres). B Close-up of the biuret binding site, with predicted hydrogen bonds displayed as dashed blue lines. The (2Fo-Fc)$_{\alpha calc}$ electron density map around biuret is displayed and contoured at 1σ. Biuret is an impurity (≤ 0.1%) of the urea that was used for soaking. **C** Omit electron density map of biuret contoured at 1σ level (green mesh). Model coordinates and experimental diffraction data have been deposited with the Protein Data Bank under accession code 8RYI (https://doi.org/10.2210/pdb8ryi/pdb).

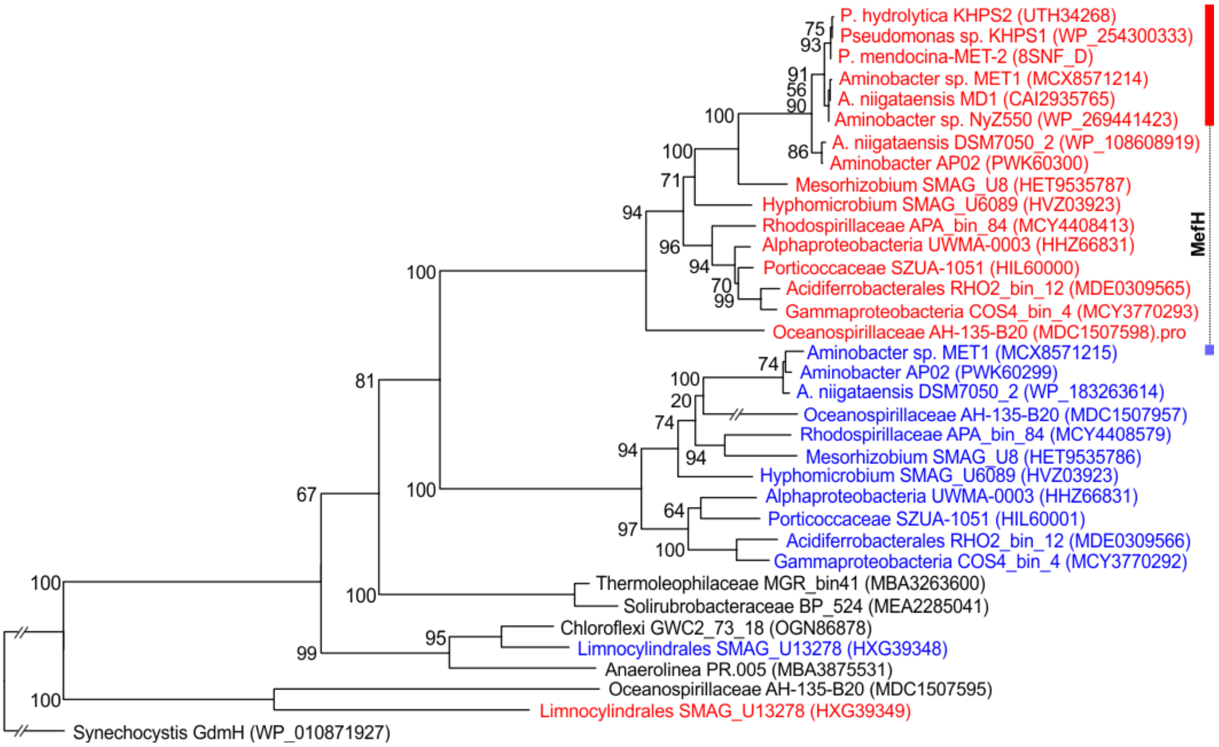

Supplementary Figure 12: Phylogenetic tree of MefH and related sequences. From BLAST searches with MefHα and MefHβ, Genbank hits representing full-length ureohydrolase sequences were selected (Note that the sequences of MefHβ are identical in all bacterial isolates that can degrade metformin). We checked in each case if they were from individual genes or from genes arranged in tandem. Red and blue letters indicate alpha and beta subunits, respectively, in tandem-arranged genes. Bootstrap values are given next to each node and NCBI protein accession numbers are in brackets behind the organism identifiers. The length of the interrupted branches was shortened for a better graphical representation of the other nodes. The trimmed sequence alignment is provided in the source data file.

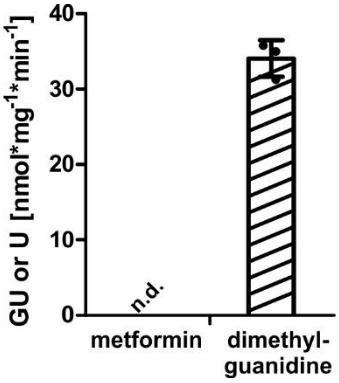

Supplementary Figure 13: Analysis of the DmgH from *Hyphomicrobium*. Bar chart showing the specific activities with 2 mM metformin or dimethylguanidine as substrates of DmgH from *Hyphomicrobium* with 70 % sequence identity for DmgHα and 68% sequence identity for DmgHβ compared to DmgH from *A. niigataensis*. Activities were determined as the substrate and enzyme-dependent production of urea (U) or guanylurea (GU). Guanylurea was determined by LC-MS and urea was determined by a colorimetric assay. Data points represent the average of triplicates and consistent results were obtained with independent preparations (n=3; error bars, s.d.). Source data are provided as a Source Data file.

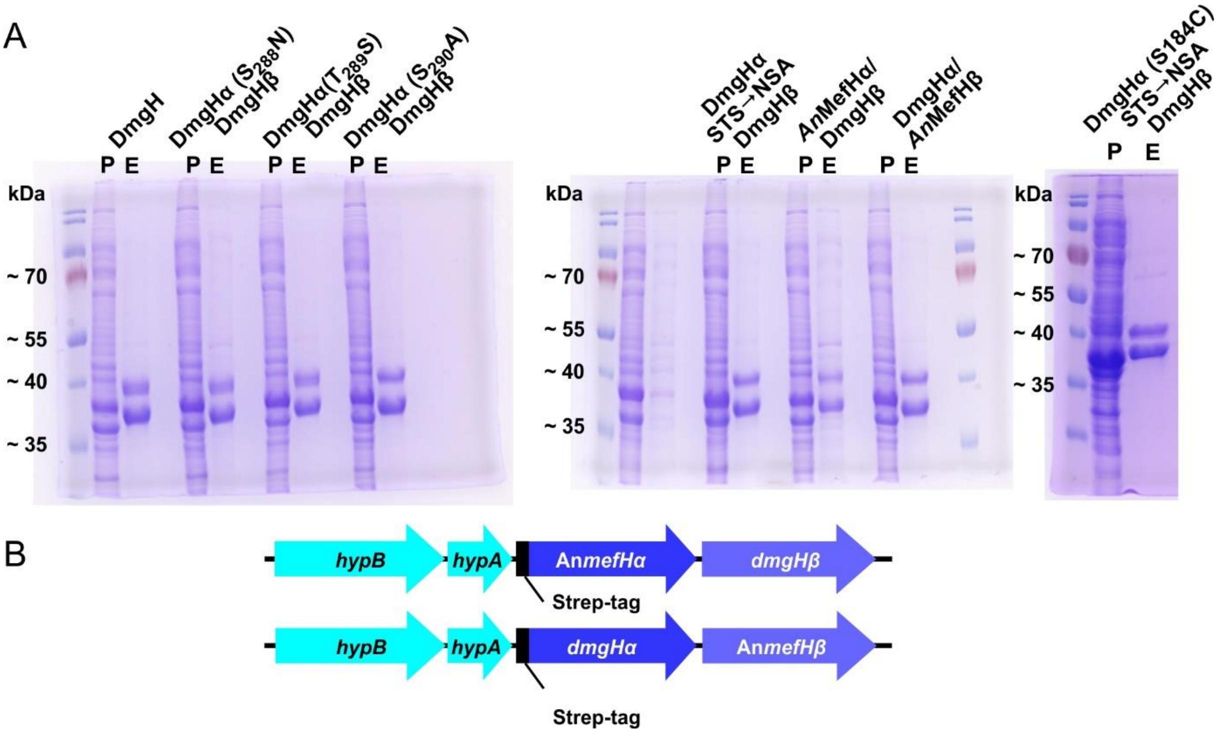

Supplementary Figure 14: Expression of MefH/DmgH mixed heteromers and DmgH variants. A Denaturing polyacrylamide gels with purified DmgH variants and mixed heteromers. **B** Schematic representation of the expression constructs for the mixed heteromers. An uncropped version of the right SDS-PA gel image can be found as Supplementary Figure 18.

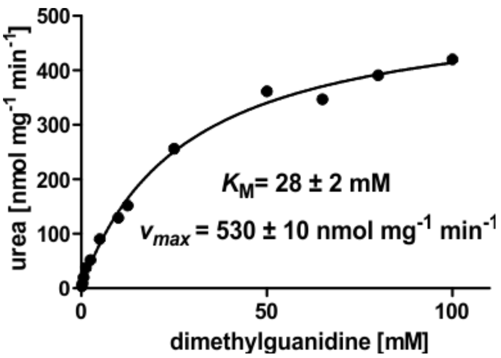

Supplementary Figure 15: Enzymatic activity of DgmH variant T289S. Urea production at substrate concentrations from 0.16-100 mM was measured to determine Michaelis constant K_M and maximal reaction rate (v_{max}) for the mutated DmgH. Data points represent the average of triplicates and consistent results were obtained with independent preparations (n=3; error bars, s.d.). Source data are provided as a Source Data file.

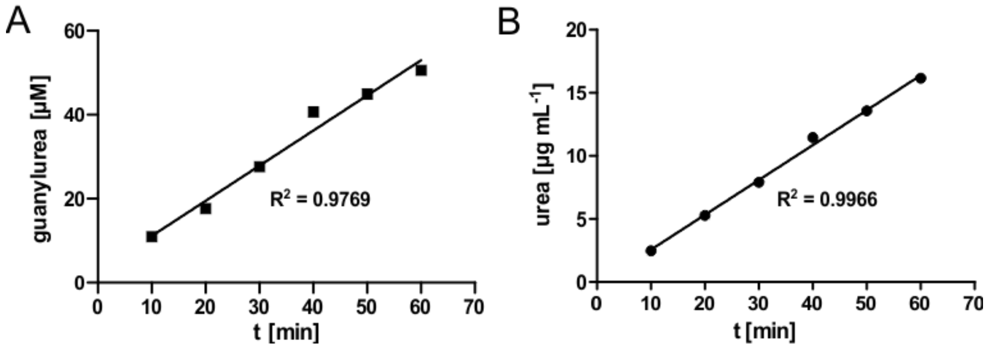

Supplementary Figure 16: Linearity of guanylurea and urea production over time. Concentrations of guanylurea (**A**) and urea (**B**) were plotted against the reaction time of the enzyme assays with MefH and DmgH, respectively. Data were analyzed by linear regression and the calculated R^2 values confirmed that the reaction was *bona fide* linear within the 1 h used in end-point determinations of enzyme activity (n=1). Source data are provided as a Source Data file.

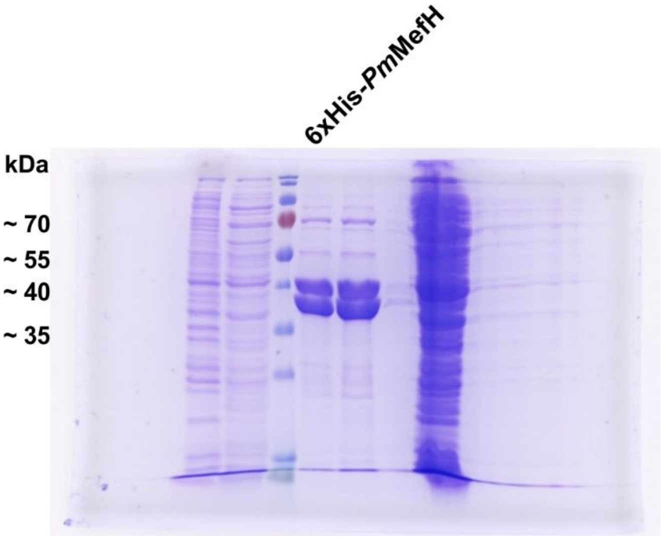

Supplementary Figure 17: Uncropped picture of the denaturing polyacrylamide gel gel shown in Supplementary Figure 2.

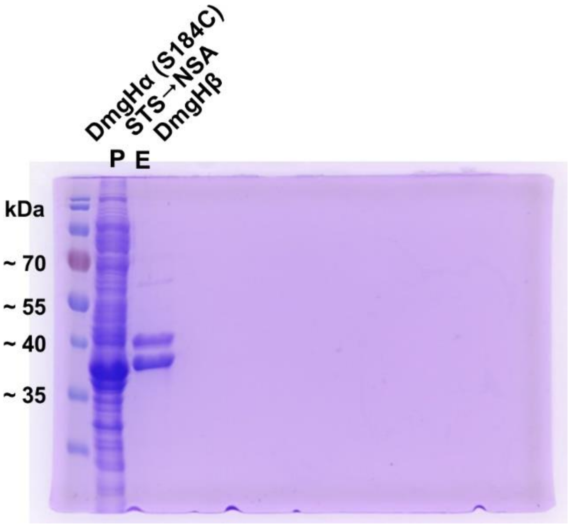

Supplementary Figure 18: Uncropped picture of the denaturing polyacrylamide gel gel shown in Supplementary Figure 14.

Supplementary Table 1: Peptide mass fingerprint analysis of purified *Pm*MefH. 6xHis-*Pm*MefH was purified by nickel affinity chromatography. Protein bands at ~37 kDa and ~42 kDa were excised from a denaturing polyacrylamide gel and subjected to peptide mass fingerprint analysis.

Band	Description	Score	Coverage	# Proteins	# Unique Peptides	# Peptides	# PSMs
**42 kDa**	MefHα	11598.23	75.20	1	23	23	428
	MefHβ	7412.98	88.22	1	27	27	376
	HypB	2392.28	68.73	1	19	19	73
**37 kDa**	MefHα	2026.02	53.07	1	16	16	84
	MefHβ	32200.81	96.26	1	35	35	1220
	HypB	5702.26	68.73	1	21	21	167

Supplementary Table 2: Compounds of the substrate screen.

metformin	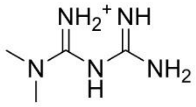	guanidine	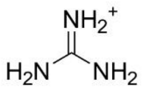
dimethylguanidine	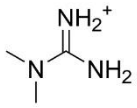	arginine	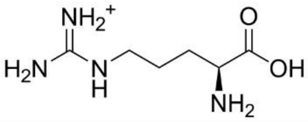
methylguanidine	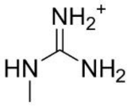	homoarginine	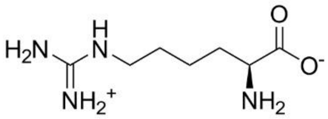
creatine	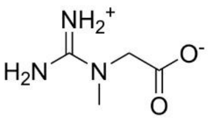	guanidinobutyrate	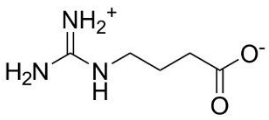
creatinine	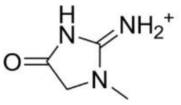	guanidinopropionate	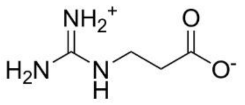
assymetric dimethylarginine	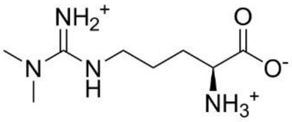	guanidinoacetate	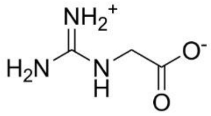
mono-methylarginine	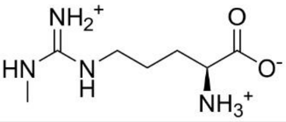	taurocyamine	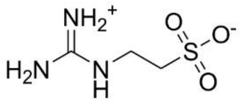

Supplementary Table 3: X-ray diffraction data collection and refinement statistics

	Metformin hydrolase
PDB code	8RYI
Space group	*C*2
Cell dimensions:	
a, b, c (Å)<br>α, β, γ (°)	163.99, 86.52, 168.41<br>90, 113.99, 90
Subunit copies in ASU	2α, 4β
**Data Processing**	
Beamline	P13 (MX1), PETRA III
Detector	Dectris EIGER1 Si 16M
Resolution (Å)	29.97 – 2.06 (2.08-2.06)[a]
No. unique reflections	130397 (3627) [a]
R_{sym}(I) (%)	11.9 (215.0) [a]
<I/σ(I)>	10.31 (0.92) [a]
$CC_{1/2}$ (%)	99.8 (35.7) [a]
Completeness (%)	97.9 (97.8) [a]
Multiplicity	7.18 (7.47) [a]
**Model Refinement**	
No. working/free reflections	130397 / 6573
R_{work}/Rf_{ree} (%)	16.17/19.53
No. Atoms Protein	15885
No. Atoms Solvent	929 x H_2O; 4 x Ni^{2+}; 2 x URE [b]; 2 x C5J [c]
B-factors Protein	62.41
B-factors ligand/ion	54.91
B-factors water	56.48
R.m.s.d. bond length (Å)	0.007
R.m.s.d. angles (°)	0.88

[a] values in parentheses correspond to the highest resolution shell; [b] URE, Urea; [c] C5J, Biuret

Supplementary References:

1 Pettersen, E. F. *et al.* UCSF Chimera--a visualization system for exploratory research and analysis. *J Comput Chem* **25**, 1605-1612 (2004). https://doi.org/10.1002/jcc.20084
