# Supplementary material for: Metformin hydrolase is a recently evolved nickel-dependent heteromeric ureohydrolase
Source: Nat Commun. 2024 Sep 14;15:8045. doi: 10.1038/s41467-024-51752-5 (PMC11399263; doi:10.1038/s41467-024-51752-5)
Supplement: Supplementary file 3 — Description of Additional Supplementary Files [file 41467_2024_51752_MOESM3_ESM.pdf]

Description of Additional Supplementary Files

File Name: Supplementary Data 1
Description: Oligonucleotides and primers used in the study
