# Supplementary material for: Metformin hydrolase is a recently evolved nickel-dependent heteromeric ureohydrolase
Source: Nat Commun. 2024 Sep 14;15:8045. doi: 10.1038/s41467-024-51752-5 (PMC11399263; doi:10.1038/s41467-024-51752-5)
Supplement: Supplementary file 5 — Reporting Summary [file 41467_2024_51752_MOESM5_ESM.pdf]

nature portfolio

Corresponding author(s): Jörg S. Hartig
Malte Sinn

Last updated by author(s): Aug 6, 2024

Reporting Summary

Nature Portfolio wishes to improve the reproducibility of the work that we publish. This form provides structure for consistency and transparency in reporting. For further information on Nature Portfolio policies, see our Editorial Policies and the Editorial Policy Checklist.

Statistics

For all statistical analyses, confirm that the following items are present in the figure legend, table legend, main text, or Methods section.

n/a	Confirmed	
☐	☒	The exact sample size (*n*) for each experimental group/condition, given as a discrete number and unit of measurement
☐	☒	A statement on whether measurements were taken from distinct samples or whether the same sample was measured repeatedly
☒	☐	The statistical test(s) used AND whether they are one- or two-sided<br>*Only common tests should be described solely by name; describe more complex techniques in the Methods section.*
☒	☐	A description of all covariates tested
☐	☒	A description of any assumptions or corrections, such as tests of normality and adjustment for multiple comparisons
☐	☒	A full description of the statistical parameters including central tendency (e.g. means) or other basic estimates (e.g. regression coefficient) AND variation (e.g. standard deviation) or associated estimates of uncertainty (e.g. confidence intervals)
☒	☐	For null hypothesis testing, the test statistic (e.g. *F*, *t*, *r*) with confidence intervals, effect sizes, degrees of freedom and *P* value noted<br>*Give P values as exact values whenever suitable.*
☒	☐	For Bayesian analysis, information on the choice of priors and Markov chain Monte Carlo settings
☒	☐	For hierarchical and complex designs, identification of the appropriate level for tests and full reporting of outcomes
☒	☐	Estimates of effect sizes (e.g. Cohen's *d*, Pearson's *r*), indicating how they were calculated

Our web collection on statistics for biologists contains articles on many of the points above.

Software and code

Policy information about availability of computer code

Data collection	Blastp search against the NCBI database
Data analysis	Enzyme Kinetics: GraphPad Prism v5.01 software. Protein Modelling: ColabFold v1.5.5 using AlphaFold2, Crystallography: PHENIX version 1.20.1_4487 which included PHASER, XDS/XSCALE (2021 release), COOT (0.8.9.1), MolProbity (http://molprobity.biochem.duke.edu, accessed in 2021). Molecular Images: UCSF ChimeraX v1.7.1 Multiple Sequence Alignment: Jalview 2.11.0 using Clustal Omega. Trimming: BioEdit. Phylogenetic tree: IQ-tree (http://iqtree.cibiv.univie.ac.at/), visualized with TreeViewer. Dynamic Light Scattering: Zetasizer Nano ZSP Software, Malvern Panalytical

For manuscripts utilizing custom algorithms or software that are central to the research but not yet described in published literature, software must be made available to editors and reviewers. We strongly encourage code deposition in a community repository (e.g. GitHub). See the Nature Portfolio guidelines for submitting code & software for further information.

Data

Policy information about availability of data

All manuscripts must include a data availability statement. This statement should provide the following information, where applicable:

- Accession codes, unique identifiers, or web links for publicly available datasets
- A description of any restrictions on data availability
- For clinical datasets or third party data, please ensure that the statement adheres to our policy

All data generated in this study are provided in the Source Data file. Model coordinates and experimental diffraction data have been deposited with the Protein

Data Bank under accession code 8RYI (https://doi.org/10.2210/pdb8ryi/pdb). X-ray diffraction images are deposited at https://doi.org/10.5281/zenodo.10638641.

Research involving human participants, their data, or biological material

Policy information about studies with human participants or human data. See also policy information about sex, gender (identity/presentation), and sexual orientation and race, ethnicity and racism.

Reporting on sex and gender	n.a.
Reporting on race, ethnicity, or other socially relevant groupings	n.a.
Population characteristics	n.a.
Recruitment	n.a.
Ethics oversight	n.a.

Note that full information on the approval of the study protocol must also be provided in the manuscript.

Field-specific reporting

Please select the one below that is the best fit for your research. If you are not sure, read the appropriate sections before making your selection.

☒ Life sciences ☐ Behavioural & social sciences ☐ Ecological, evolutionary & environmental sciences

For a reference copy of the document with all sections, see nature.com/documents/nr-reporting-summary-flat.pdf

Life sciences study design

All studies must disclose on these points even when the disclosure is negative.

Sample size	Sample sizes were not calculated in advance becaue no complex statistical analyses were intended. Biochemical experiments were conducted at least in duplicates. Biochemical experiments were repeated in biological replicates (see below). Thus we ensured reproducibilty of the enzyme assays with indepenndet enzyme preparations and showed reliabilty of our procedure. Wherever three replicates of enzyme were performed the data were used to calculate s.d.
Data exclusions	Single data points from obviously erroneously composed enzyme assays were excluded.
Replication	Experiments were replicated at least once, results were consistent throughout the experiments.
Randomization	Randomization would not contribute to data reliability.
Blinding	None of the applied methods of measurement and analysis are susceptible to personal prejudice, therefore no blinding was applied.

Reporting for specific materials, systems and methods

We require information from authors about some types of materials, experimental systems and methods used in many studies. Here, indicate whether each material, system or method listed is relevant to your study. If you are not sure if a list item applies to your research, read the appropriate section before selecting a response.

Materials & experimental systems

n/a	Involved in the study	
☒	☐	Antibodies
☒	☐	Eukaryotic cell lines
☒	☐	Palaeontology and archaeology
☒	☐	Animals and other organisms
☒	☐	Clinical data
☒	☐	Dual use research of concern
☒	☐	Plants

Methods

n/a	Involved in the study	
☒	☐	ChIP-seq
☒	☐	Flow cytometry
☒	☐	MRI-based neuroimaging

Plants

Seed stocks	n.a.
Novel plant genotypes	n.a.
Authentication	n.a.
